# Efficient Manipulation of Continuous AFI-Type Aluminophosphate Membranes with Distinctive Microstructures on Macroporous α-Al_2_O_3_ Substrates

**DOI:** 10.3390/molecules23051127

**Published:** 2018-05-09

**Authors:** Luwei Geng, Hongfeng Dong, Xiufeng Liu, Baoquan Zhang

**Affiliations:** School of Chemical Engineering and Technology, Tianjin University, Tianjin 300350, China; gengluwei@163.com (L.G.); xfliu@tju.edu.cn (X.L.); bqzhang@tju.edu.cn (B.Z.)

**Keywords:** AFI-type aluminophosphate, oriented membrane, chitosan, microstructure manipulation

## Abstract

The availability of continuous and well-defined AFI-type aluminophosphate membranes (AFI membranes) would trigger their applications in innovative materials. A well-designed manipulation strategy is proposed to produce continuous AFI membranes with four different microstructures over porous α-Al_2_O_3_ substrates. A double-layer and highly *c*-oriented AFI membrane of hexagonal prisms is obtained when a thin layer of medium molecular weight (MMW) chitosan is employed as the structure-directing matrix together with aluminum isopropoxide (AIP) as the Al source. It can be transformed to a single-layer and highly *c*-oriented AFI membrane of hexagonal prisms if the structure-directing matrix is replaced by a thin layer of low molecular weight (LMW) chitosan. When the Al source is changed to pseudo-boehmite, the single-layer AFI membrane is composed of highly ordered spherical agglomerates of small crystals. Furthermore, the membrane will turn to the double-layer AFI membrane of highly-ordered crystal agglomerates if a thin layer of MMW chitosan is used once again, keeping pseudo-boehmite as the Al source. The manipulation methodology established here is rather reliable with a pretty high reproducibility.

## 1. Introduction

Microporous crystalline materials, mainly including zeolites and aluminophosphate molecular sieves, have attracted extensive interests in many fields such as adsorption, catalysis, ion exchange, microelectronics, and chemical sensing due to their large specific surface areas, defined channel systems, high mechanical strength, and hydrothermal stability. Zeolites containing cages and pores of molecular dimensions, which give them their size and shape selectivity, have received intense research for over 60 years [1]. Furthermore, aluminophosphate molecular sieves (AlPO-n), which are built of strictly alternating [AlO_4_] and [PO_4_] tetrahedral units, form a new class of microporous inorganic solids. Following the initial report of aluminophosphates in 1982 [2], the isomorphous substitution of aluminum and phosphorus in the framework with silicon and metals were successful in 1984 and 1986, respectively [3,4]. Aluminophosphates exhibit both compositional and structural diversities. The structural features and unique physicochemical properties of microporous aluminophosphates have expanded the application area of microporous materials [5]. The AFI-type molecular sieve (AlPO4-5 with derivatives), which was composed of one-dimensional channels with a pore diameter of 0.73 nm aligned parallel to its crystallographic *c*-axis, is one of the most and earliest studied members in the family of aluminophosphates [5,6].

Zeolite and molecular sieve membranes with uniform and molecular-sized pores have excellent thermal, mechanical, and chemical stabilities, and they have acquired potential applications as separation membranes and membrane catalysts [7,8,9,10,11]. Besides, new application areas such as low-dielectric constant materials as well as anti-corrosion and anti-microbial coatings have been developed [12,13]. The microstructure of zeolite and molecular sieve membranes including continuity, crystal orientation, and thickness is of great significance in actual applications [8,14]. For instance, the denseness of a molecular sieve membrane is essential for the realization of molecular-sieving function. The zeolite membrane with a preferential crystal orientation shows a greatly improved performance. Ultrathin membranes possess significant advantages that improve permeation performance, while thick membranes prolong the mean residence time of reactants within the catalysis membrane to increase the reaction conversion. However, manipulating the microstructure of zeolite membranes is still a challenging issue.

The synthesis of AFI-type aluminophosphate membranes (AFI membranes) has been focused on controlling the crystal orientation to achieve c-oriented channels perpendicular to the substrate surface, leading to the uniform residence time of involved molecules across diffusion channels. Up to the present, there have been several routes for fabricating AFI arrays, films, and membranes with a preferred orientation, including electric field-driven assembly [15,16,17,18,19], geometry-confined growth [20,21,22], epitaxial growth [23,24,25,26], the manipulation of synthesis conditions [19,27,28,29], microwave-enhanced growth [6,30,31], and induced growth at the substrate surface [32,33].

The highly oriented AFI arrays and films were produced by the electric field-driven assembly or the geometry-confined growth, which could be used as the seed layers in the following seeded growth step to make continuous, dense, and preferentially *c*-oriented AFI membranes [19,21,22]. Besides, the array or film that was synthesized as such had to be fixed to the substrate surface using other materials before actual applications [16,17]. Basically, the availability of oriented seed layers would be a prerequisite to the epitaxial growth [34]. The manipulation of synthesis conditions was a commonly used method, which required a great deal of experimental investigations [35]. If the growth mechanism and critical influence factors were clear, the cumbersome experiments could be substantially reduced. The manipulation of synthesis conditions could be further enhanced by using microwave heating, which had been applied to either the in situ crystallization [6] or the seeded growth [30,31]. It should be noted that the majority of the AFI membranes that have been reported to date were prepared on dense substrates such as silicon wafers or glass plates, which would not be usable in separation and catalysis processes.

By the surface induction, the *c*-oriented AlPO4-5 crystal grains were attached onto the zirconium phosphonate-modified gold surface because of the strong affinity of phosphonic acid groups to the (*00l*) planes [32]. In our previous report [33], a precoated chitosan thin layer on a macroporous α-Al_2_O_3_ substrate was used to act as the orientation directing matrix. After the in situ crystallization, the well intergrown and preferentially *c*-oriented SAPO-5 films over a closely packed layer of SAPO-5 crystals was formed, accompanied with the gradual dissolution of chitosan. Herein, we wish to further manipulate the microstructure of AFI membranes in the in situ crystallization. As such, continuous and dense AFI membranes with single and double-layered structures covered with either highly *c*-oriented hexagonal prisms or spherical agglomerates could be synthesized at will. There are not relevant reports yet.

## 2. Results

### 2.1. Double-Layer and c-Oriented AFI Membranes

**Figure 1:**
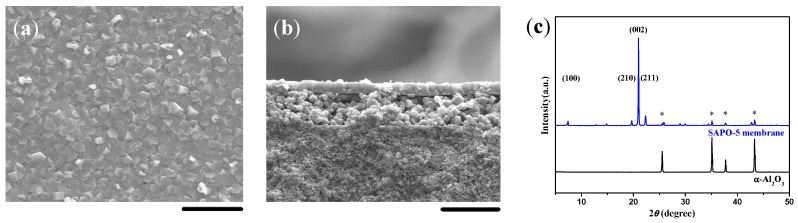
(**a**) Top view and (**b**) cross-sectional SEM images together with (**c**) the corresponding XRD pattern for the double-layer SAPO-5 membrane induced by a medium molecular weight (MMW) chitosan layer using aluminum isopropoxide (AIP) as the Al source. Scale bars correspond to 20 μm in SEM images. The asterisks in XRD patterns mark the peaks originating from the α-Al_2_O_3_ substrate.

In our previous work, a thin layer of MMW chitosan was coated on the α-Al_2_O_3_ substrate to induce the formation of a double-layer SAPO-5 membrane, i.e., a highly *c*-oriented and well intergrown SAPO-5 top layer over a closely packed bottom layer of SAPO-5 crystals (Figure 1). After the *c*-oriented SAPO-5 crystals on the top layer were completely formed, the precursor solution was unable to penetrate into the bottom layer, leading to the formation of the double-layer SAPO-5 membrane on the α-Al_2_O_3_ substrate. It should be noted that the continuity and thickness of the highly *c*-oriented SAPO-5 top layer and the packed bottom layer could be optimized by regulating the ingredients of the synthesis solution and the substrate setting.

In the following studies, the optimized substrate setting (facing downwards) and the SiO_2_/Al_2_O_3_ ratio in the synthesis solution (0.15) would be adopted unless otherwise stated. Due to the gradual dissolution of chitosan in the in situ crystallization, both the surface and entire body of the chitosan layer would serve as a three-dimensional structure-directing matrix. The dissolution rate of chitosan ought to be a key factor to influence the formation of SAPO-5 membranes with a unique microstructure on porous α-Al_2_O_3_ substrates.

### 2.2. Single-Layer and c-Oriented AFI Membranes

According to a literature report [36], a chitosan tablet usually swelled from the surface to its inner part in acidic or neutral media while the internal glassy region would decline until the glassy core vanished. All swollen gels would dissolve gradually. Compared with low molecular weight (LMW) chitosan, medium molecular weight (MMW) chitosan swelled to some deeper extent while exhibiting a low erodible rate. The dissolution rate of both LMW and MMW chitosan in the acidic solution was also checked in our experiment, the result of which was in accordance with that of Huanbutta et al. [36]. As such, we would assume a LMW chitosan coating with a thinner swelling layer and increased dissolution rate would direct the crystal growth another way, leading to the formation of an AFI membrane with a novel microstructure. Therefore, LMW chitosan was employed to prepare a thin layer on the α-Al_2_O_3_ substrate to induce the growth of SAPO-5 films in the in situ crystallization.

**Figure 2:**
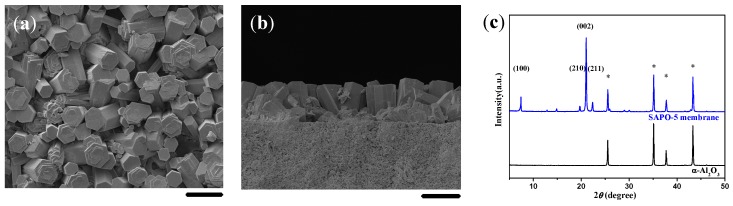
(**a**) Top view and (**b**) cross-sectional SEM images together with (**c**) the corresponding XRD pattern for the single-layer SAPO-5 membrane induced by a layer of LMW chitosan using AIP as the Al source. Scale bars correspond to 20 μm in SEM images. The asterisks in XRD patterns mark the peaks originating from the α-Al_2_O_3_ substrate.

As shown in Figure 2, a single-layer SAPO-5 film could be formed on the α-Al_2_O_3_ substrate due to the induction of the LMW chitosan layer. The SAPO-5 film was composed of hexagonal prisms, most of which were aligned normally to the substrate surface. The SAPO-5 film exhibited preferred *c*-orientation, as confirmed by the XRD pattern where the diffraction peak of (002) plane at 21.05° was much stronger. However, the compactness of the SAPO-5 film was not satisfactory at the present stage. The manipulation was further required to increase the continuity and crystal orientation of the synthesized SAPO-5 membrane.

Based on available reports in the literature [27,32,37], the effect of water content on the morphology and size of AFI-type crystal grains was quite remarkable. A high H_2_O/Al_2_O_3_ ratio in the synthesis solution favored preferential growth along the *c*-axis, resulting in AFI-type crystals with a high aspect ratio (length versus diameter). Decreasing the water content would suppress the one-dimensional growth along the *c*-axis to form blocky or even plate-like crystals. In the formation of highly *c*-oriented AFI membranes, the fast growth along the *c*-axis (vertical to the substrate surface) would slow down the in-plane growth, and restrain the intergrowth of crystal grains. A suitable value of the water content in the synthesis solution would be essential to the manipulation of the microstructure.

**Figure 3:**
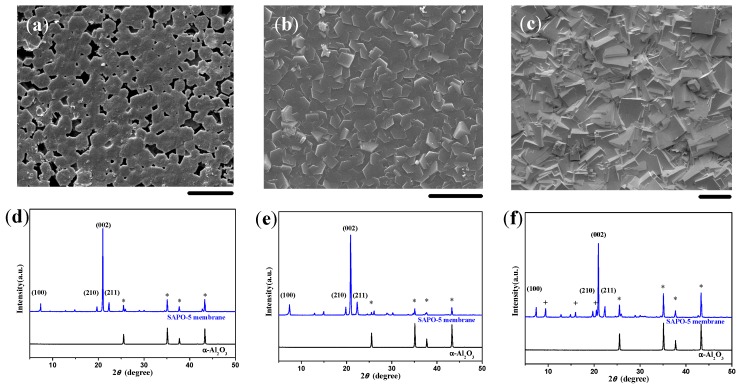
SEM images and the corresponding XRD patterns of SAPO-5 membranes induced by a layer of low molecular weight (LMW) chitosan using AIP as the Al source and the H_2_O/Al_2_O_3_ ratio of 200 (**a**,**d**), 150 (**b**,**e**), and 100 (**c**,**f**). Scale bars correspond to 20 μm in SEM images. The asterisks and plus signs mark the peaks from the α-Al_2_O_3_ substrate and SAPO-34, respectively.

When the molar ratio of H_2_O/Al_2_O_3_ in the synthesis solution was 300, the as-synthesized crystals were elongated hexagonal prisms, which arrayed upwards in general with an inclination angle and unfilled defects everywhere (Figure 2). According to the XRD pattern given in Figure 2c, the diffraction peaks of (100), (210), and (211) planes exhibited higher intensity. Moreover, the size of the crystals was relatively large, owing to the lower density of nuclei using the diluted solution. When the water content was further decreased to the level of H_2_O/Al_2_O_3_ = 200, the membrane composed of smaller and denser crystals possessed a high c-orientation and improved continuity, as shown in Figure 3a,d. A further decrease of the H_2_O/Al_2_O_3_ molar ratio to 150 resulted in a highly *c*-oriented SAPO-5 membrane where the hexagonal crystals were closely intergrown (Figure 3b,e). The membrane surface became much smoother without any crystal boundaries. When a more concentrated synthesis solution was used, the hexagonal crystals could not be observed anymore on the membrane surface, although the XRD pattern revealed a preferentially *c*-oriented membrane (Figure 3c,f). The emergence of diffraction peaks at 9.5°, 16.0°, and 20.6° in the XRD pattern (Figure 3f) together with the cubic crystals existed on the membrane surface (Figure 3c) indicated the structure transformation from SAPO-5 to SAPO-34 molecular sieves. Moreover, the enhancement of diffraction peaks of the substrates implied that the continuity of the membrane decreased in this case. Therefore, the molar ratio of H_2_O/Al_2_O_3_ = 150 should be the appropriate for the synthesis of a c-oriented SAPO-5 membrane with a single-layer microstructure in this system.

**Figure 4:**
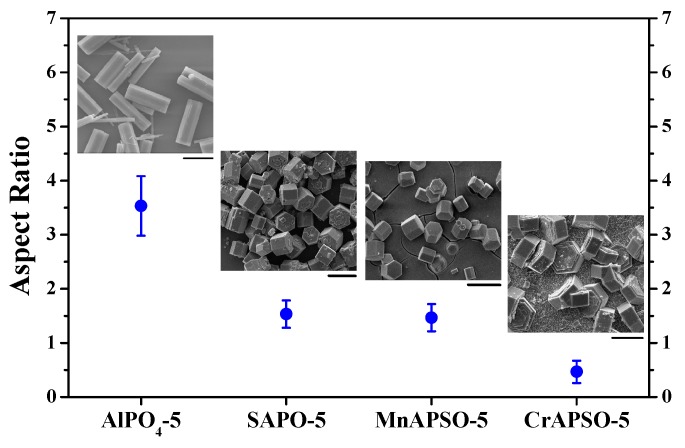
The average aspect ratios together with the corresponding size distributions for AFI molecular sieve crystals with or without heteroatoms. Scale bars correspond to 20-μm SEM images.

**Figure 5:**
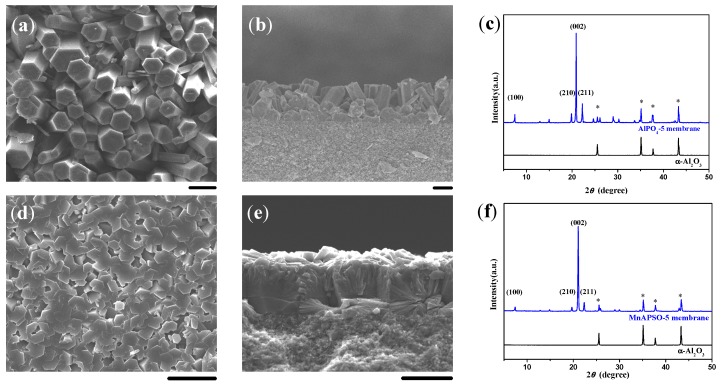
Top view and cross-sectional SEM images together with the corresponding XRD patterns for (**a**–**c**) AlPO_4_-5 and (**d**–**f**) MnAPSO-5 membranes induced by a layer of LMW chitosan using AIP as the Al source. Scale bars correspond to 20 μm in SEM images. The asterisks in the XRD patterns mark the peaks originating from the α-Al_2_O_3_ substrate.

It has been revealed that the existence of silicon species would be conducive to the formation of dense AFI membranes based on literature reports and our experiments. In the absence of silicon in the synthesis solution, AlPO4-5 molecular sieves on the substrate surface would grow much faster along the c-axis, as shown in Figure 4, resulting in elongated crystals as long as 40–50 μm. This would be detrimental to the intergrowth of crystal grains. Compared with SAPO-5 synthesized in the same conditions, AlPO4-5 molecular sieves were too slender to be arranged regularly on the substrate surface (Figure 5a,b). According to the XRD pattern (Figure 5c), the degree of c-orientation of the crystal layer was lower than the SAPO-5 membrane, and the enhancement of the diffraction peaks of the substrate suggested the discontinuity of the molecular sieve layer.

Several research groups worldwide have reported that the addition of silicon species into the reaction mixture decreases the growth rate of AFI molecular sieves along the *c*-axis, leading to crystals with low aspect ratios [38,39,40,41,42]. The growth inhibition along the *c*-axis should be beneficial to increase the in-plane growth of the AFI membrane. As a typical example, the continuous SAPO-5 membranes could be fabricated via the in situ crystallization method, but the AlPO_4_-5 analogues could not, until now. On the other hand, the isomorphous substitution of framework atoms by transitional metals could provide redox active sites on molecular sieves for heterogeneous catalysis [43]. MnAPSO-5 crystals possessed almost the same morphology and size as SAPO-5 (Figure 4). The MnAPSO-5 membrane with the thickness of 20 μm showed excellent continuity as well as a preferential *c*-orientation (Figure 5d–f).

The incorporation of chromium could further reduce the length of hexagonal prisms along the c-axis (Figure 4). Although plate-like CrAPSO-5 crystals could be synthesized with an aspect ratio of ca. 0.5, the *c*-orientation of the CrAPSO-5 membrane was not as prominent as that of the SAPO-5 and MnAPSO-5 membranes, according to the enhancement of diffraction peaks of the (100), (210), and (211) planes (Figure S1). This phenomenon suggested that the aspect ratio of crystals would not be the sole factor to influence the c-orientation of AFI membranes.

### 2.3. Single-Layered AFI Membranes of Crystal Agglomerates

**Figure 6:**
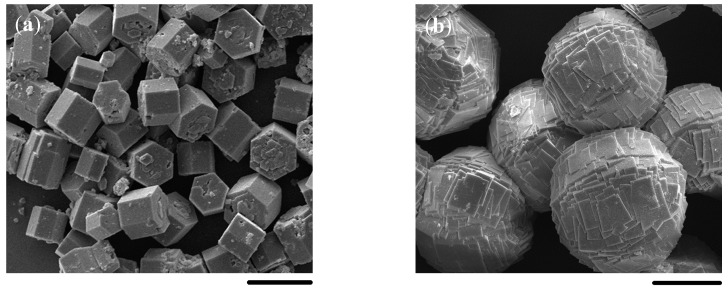
SEM images of SAPO-5 crystal grains using AIP (**a**) and pseudo-boehmite (**b**) as the Al source. Scale bars correspond to 20 μm.

The morphology of AFI crystal grains using AIP as the Al source was mainly hexagonal prisms based on our own experimental results and literature reports (Figure 6a). When pseudo-boehmite was used as the Al source, spherical crystal agglomerates would be synthesized as shown in Figure 6b. These multi-crystal and dense agglomerates were formed through the combination of a great amount of flaky crystals, which might be conducive to the formation of continuous AFI membranes.

**Figure 7:**
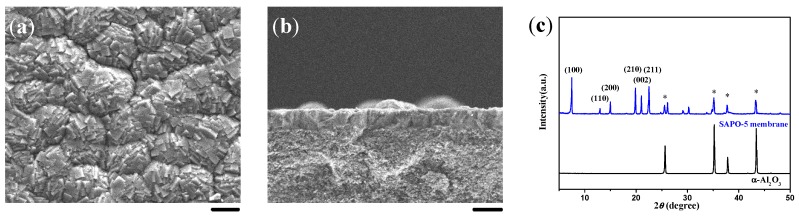

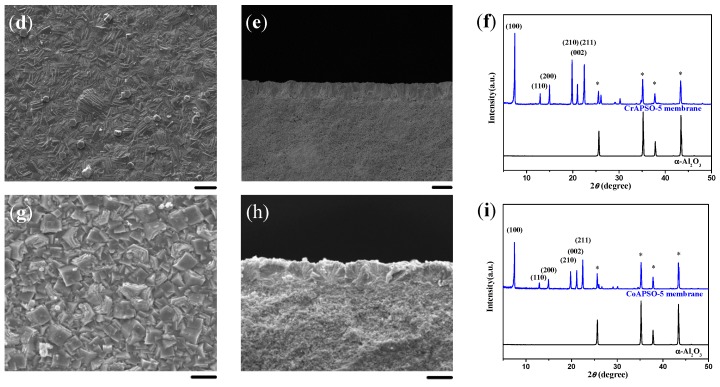
Top view and cross-sectional SEM images together with the corresponding XRD patterns for (**a**–**c**) SAPO-5, (**d**–**f**) CoAPSO-5, and (**g**–**i**) CrAPSO-5 membranes induced by a layer of LMW chitosan using pseudo-boehmite as the Al source. Scale bars correspond to 20 μm in SEM images. The asterisks in the XRD patterns mark the peaks originating from α-Al_2_O_3_ substrate.

When AIP was replaced by pseudo-boehmite as the Al source, while the other components and crystallization conditions were kept the same, the 15–20-μm thick and continuous SAPO-5 membrane composed of intergrown agglomerates could be formed (Figure 7a–c). The XRD patterns shown in Figure 7c exhibited a highly crystallized AFI-type framework without any impurities. The compactness of the membrane before template removal was checked by using the leaking test, where the permanence of N_2_ was far below 10^−9^ mol m^−2^ s^−1^ Pa^−1^ [44].

Based on a number of experimental investigations, AFI molecular sieves could be synthesized within a relatively wide range of pH using the ingredients in this study. The variation of pH would cause the morphology change of AFI crystal grains on the one hand. On the other hand, the dissolution rate of the chitosan layer should be varied under different pH values, resulting in the different microstructure of the synthesized membrane (Figure S2). At pH = 4.5, the agglomerated SAPO-5 crystals were packed loosely on the substrate surface due to a faster dissolution of the chitosan layer in the stronger acidic solution, where the structure-directing function of the chitosan layer should be very weak (Figure S2a,b). When the pH of the synthesis solution was changed to 6.0, the intergrowth of AFI crystals in the as-synthesized SAPO-5 membrane was significantly improved (Figure S2c,d). If the pH was further changed to 7.0, a continuous layer of crystal agglomerates could not be formed on the substrate surface. Some chitosan residuals could be observed on the membrane surface (Figure S2e). It should be noted that the dissolution rate of chitosan was low in the neutral synthesis solution compared with the above two situations. Actually, this phenomenon acted as another validation for the relationship between the dissolution rate and the microstructure of AFI membranes.

As aforementioned, a number of transitional metal ions could be incorporated into the framework of AFI molecular sieves for various potential applications [43,45]. Several metallic elements such as Cr, Co, Fe, and Mn were added into the reaction systems to synthesize MeAPSO-5 membranes in this study. As shown in Figure 7d–i and Figure S3, the effect of incorporated transitional metals on the morphology of crystal agglomerates was very tiny, and single-layered MeAPSO-5 membranes with satisfactory denseness could be obtained under the structure-directing function of a LMW chitosan layer.

**Figure 8:**
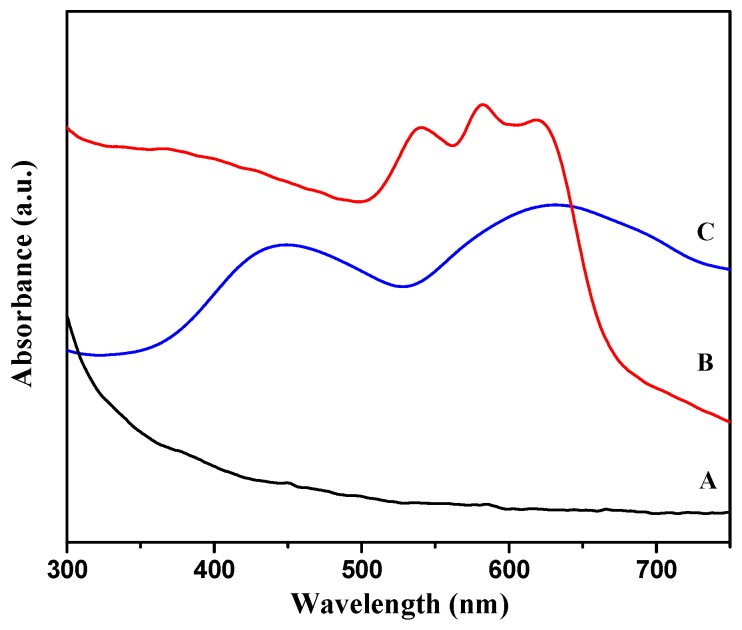
Diffuse-reflectance UV–vis spectra of (A) SAPO-5, (B) CoAPSO-5, and (C) CrAPSO-5 membranes corresponding to Figure 7.

Diffuse Reflectance UV-vis spectroscopy could provide an evidence of the incorporation of cobalt and chromium into the AFI framework. The triplet bands located in the 530–650-nm region were present only in the CoAPSO-5 membrane (Figure 8, line B), which was attributed to the d–d transitions of tetrahedrally coordinated Co^2+^ [28,46,47]. There was still a controversy about the attribution of absorption peaks to the framework substitution of chromium, the bands appearing at 440 nm and 620 nm for the as-synthesized CrAPSO-5 membrane were generally recognized as the feature of Cr(III) in distorted octahedral coordination (Figure 8, line C), where the Cr(III) heteroatoms bonded tetrahedrally to the framework were complementarily coordinated by two H_2_O molecules as additional ligands [48,49,50]. In addition, the blue and green color of the synthesized molecular sieves also indicated that both Co^2+^ and Cr^3+^ had been incorporated into the AFI framework, respectively.

The contents of P, Al, Si, and metals along the cross-section of an AFI membrane were measured by using the electron probe microanalysis (EPMA) technique (Figure S4). In accordance with the composition in the synthesis solution, the concentration of either P or Al was high, while Si and metal (Fe or Mn) was sparsely distributed within the membrane.

### 2.4. Double-Layered AFI Membranes of Crystal Agglomerates

**Figure 9:**
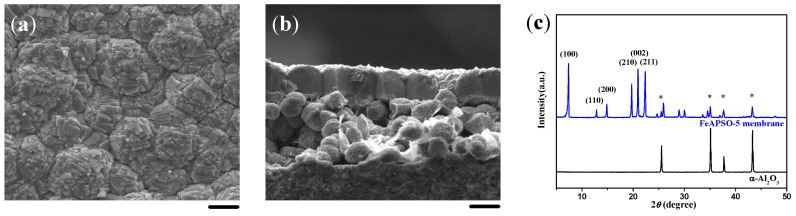
(**a**) Top view and (**b**) cross-sectional SEM images together with (**c**) the corresponding XRD pattern of the double-layer FeAPSO-5 membrane induced by a layer of MMW chitosan using pseudo-boehmite as the Al source. Scale bars correspond to 20 μm. The asterisks in XRD patterns mark the peaks originating from the α-Al_2_O_3_ substrate.

When a thin layer of MMW chitosan was covered on the substrate surface while pseudo-boehmite was used as the Al source, the double-layered MeAPSO-5 membranes of crystal agglomerates were obtained (Figure 9 and Figure S5). No matter whether Fe (Figure 9), Mn (Figure S5a,b), or Co (Figure S5c,d) was incorporated, a 40–50 μm-thick bottom layer consisted of loosely packed spherical particles, while the top layer was 20–50 μm-thick and consisted of a well-intergrown membrane of crystal agglomerates. The dense MeAPSO-5 top layer was different from the *c*-oriented AFI membrane of hexagonal prisms that was synthesized by using AIP as the Al source. The XRD patterns indicated that the membrane was composed of AFI crystals with high crystallinity without any impurities. The attenuation of diffraction peaks of the substrate suggested the α-Al_2_O_3_ was fully covered by the double-layered AFI membrane of crystal agglomerates.

**Figure 10:**
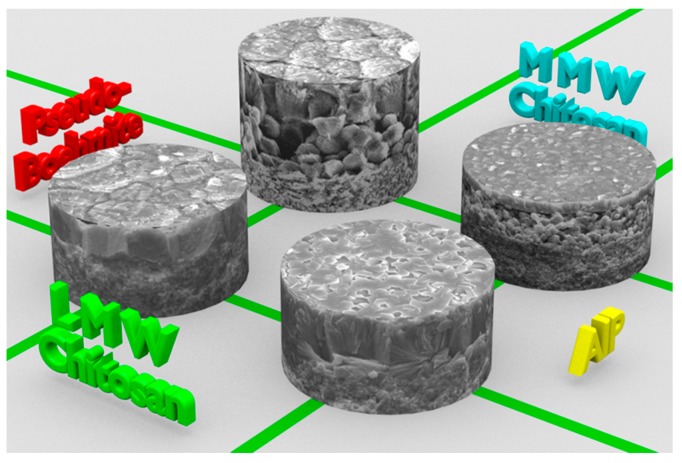
Schematic diagram of manipulating microstructure of AFI membranes using a thin chitosan layer of different molecular weight and different Al sources.

Obviously, when the Al source was changed from pseudo-boehmite back to AIP, and the other components were kept the same as that of Zhang et al. [33], the synthesized product would be the double-layered AFI membrane as shown in Figure 1, where the bottom layer was made up of loosely packed spherical particles, and the top layer was composed of well-intergrown and *c*-oriented hexagonal prisms. Thus, a close circuit should be finally formed by changing the structure-directing matrix (a thin layer of chitosan with different molecular weight) and the Al source (AIP and pseudo-boehmite) to achieve the microstructure manipulation of AFI membranes with high reproducibility (Figure 10).

## 3. Formation Mechanism

**Figure 11:**
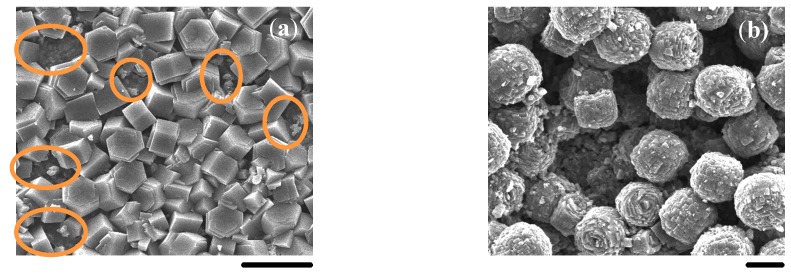
SEM images of SAPO-5 layers synthesized on unmodified α-Al_2_O_3_ substrates using AIP (**a**) and pseudo-boehmite (**b**) as the Al source. Scale bars correspond to 20 μm.

A randomly oriented and compact SAPO-5 membrane could be fabricated on an unmodified α-Al_2_O_3_ substrate by using AIP as the Al source under microwave heating [6]. However, the synthesized SAPO-5 film of hexagonal prisms on the unmodified α-Al_2_O_3_ substrate was both randomly oriented and non-continuous using traditional heating (Figure 11a), which was in full agreement with our previous report [33]. Voids or defects between hexagonal crystals spread all over the substrate surface (circled areas). By using pseudo-boehmite as the Al source, only some bigger crystal agglomerates were dispersed on the unmodified α-Al_2_O_3_ substrate with much larger voids and open areas under traditional heating (Figure 11b). Control experiments using bare α-Al_2_O_3_ substrates as indicated above confirmed that the chitosan layer should be indispensable for the continuity and the *c*-orientation of AFI membranes on porous α-Al_2_O_3_ substrates in the in situ crystallization.

The research on the formation mechanism of supported zeolite films and membranes dates back to the late 1990s [51,52]. According to available literature reports, there are two formation mechanisms named the heterogeneous nucleation model (HTN model) and the homogeneous nucleation model (HMN model). In the presence of a chitosan layer, a gel layer is initially formed on the supported chitosan surface in the in situ crystallization. Both nucleation and crystal growth occur inside the gel layer and in the bulk synthesis solution as well. Whether the HTN or HMN model applies to the film formation is directly related to the ratio of the two growth rates [52,53].

Chitosan is insoluble in water and aqueous basic media, but it becomes soluble in aqueous acidic media at pH < 6.5 [54]. Since MMW chitosan heavily swelled with a low erodible rate [38], the entire chitosan layer functioned as a three-dimensional (3-D) structure-directing matrix in the in situ crystallization, leading to a five-step chronological evolution of double-layer AFI membranes, as given in our previous report (Figure S6) [33]. The chitosan layer swelled with dissolution (or was being eroded) at first (A2). In the following, the crystal nucleation occurred in the bulk synthesis solution, in the eroded pores, and on the outer surface of the chitosan layer (A3). The crystals on the top layer at the outer surface and the packed crystals underneath would grow simultaneously (A4 and A5). When the top layer became well intergrown, it would act as a barrier between the precursor solution and the packed crystals, eventually leading to the double-layer AFI membranes, as shown in Figure S6 (A6).

Similar to the above, the precoated thin layer of LMW chitosan disappeared after the hydrothermal reaction. Based on the literature report [36] and our own experiment, a layer of LMW chitosan had an increased dissolution rate, but its swelling extent was reduced compared with MMW chitosan. When LMW chitosan was applied, the chitosan layer dissolved to the bulk solution faster, and the precursor solution would contact the chitosan layer all of the time, leading to the formation of the single-layer AFI membranes at last. Therefore, the chronological evolution of singer-layer AFI membranes over porous α-Al_2_O_3_ substrates under the structure-directing effect of the LMW chitosan layer could be generally defined in five steps along the elapsed time of hydrothermal reaction as follows (Figure S7): (1) the onset of chitosan swelling and its dissolution in an increased rate, where the chitosan layer was getting thinner from B1 to B2; (2) the crystal nucleation in the bulk precursor solution, in the eroded pores as well as on the outer surface of the chitosan layer, accompanied by an ever-reducing layer thickness (B3); (3) the growth of nucleus in the eroded pores as well as on the surface of the chitosan layer (B4); (4) the growth of small crystals to be intergrown on the substrate surface due to the disappearance of the chitosan layer (B5); and (5) the oriented growth of the AFI membrane after it has been well intergrown (B6).

## 4. Materials and Methods

The synthesis procedure of AFI membranes on porous α-Al_2_O_3_ substrates is similar to that reported in our previous work [33], which usually included (1) the treatment of substrates; (2) the preparation of chitosan layers; (3) the synthesis of precursor gels; and (4) the hydrothermal crystallization of AFI membranes and sample cleaning.

The homemade porous α-Al_2_O_3_ substrates with a thickness of 2 mm and a diameter of 20 mm were polished with sandpaper and sequentially treated with 1 M HCl and 1 M NaOH solution, followed by ultrasonic treatment in acetone and dried in clean atmosphere overnight.

The substrates were spin-coated with 2 wt.% chitosan solution, which was made by dissolving 75–85% deacetylated chitosan with medium molecular weight (MMW chitosan, 150–240 kDa, Aldrich, St. Louis, MO, USA) or low molecular weight (LMW chitosan, 50–190 kDa, Aldrich) in a ca. 2 wt.% acetic acid solution. The coating was performed at a spinning rate of 1600 rpm for 60 s using a spin-coater (KW-4A, Microelectronic Institute of Chinese Academy of Sciences, Beijing, China). After being coated three times, the α-Al_2_O_3_ supported chitosan layer was dried at room temperature overnight. Before use, it was refluxed in ethanol for 30 min to remove possible residues, and dried at 393 K for 30 min.

The precursor sol was prepared as follows. Aluminum isopropoxide (AIP, 99.5%, Aldrich) was hydrolyzed in deionized water at 353 K using a water bath heating under stirring for 12 h. After cooling down naturally, orthophosphoric acid (H_3_PO_4_, 85%) was added into the synthesized alumina sol dropwise under stirring at 298 K. Then, the solution was stirred for 10 h to form a white uniform gelation. The aqueous solution of metal salts was added accordingly if necessary. Triethylamine (TEA, 99%, Tianjin Chemical Reagent Co., Inc., Tianjin, China) and tetraethylorthosilicate (TEOS, 98%, Aldrich) were added sequentially under vigorous stirring, followed by aging for 12 h at room temperature. The final molar ratio of the synthesis solution was Al_2_O_3_:P_2_O_5_:0.15SiO_2_:2.4TEA:(100–300)H_2_O(:0.02 MeO_x_). At last, the pH of the sol was adjusted to ca. 6 using acetic acid (99.5%, Tianjin Chemical Reagent Co., Inc.).

When pseudo-boehmite was used as the Al source, the hydrolyzation step could be omitted. Pseudo-boehmite was added into the H_3_PO_4_ solution, stirring for 12 h. The steps afterwards were nearly the same as the above except that the pH of the solution was adjusted to 4.0–8.0.

The precursor solution (ca. 35 mL) was loaded into a 60-mL Teflon-lined stainless steel autoclave, where the chitosan-modified substrate was placed in the autoclave with the treated side facing down to the bottom. The hydrothermal treatment was performed in the sealed autoclave at 453 K for 72 h. After the autoclave was cooled down, the sample was removed and washed with DI water, and dried at 333 K overnight. The crystal grains at the bottom of the autoclave were collected through centrifugation and washing repeatedly.

The compactness of each membrane was tested by gas permeation using a leaking-test setup equipped with a soap film gas flowmeter and a gas chromatograph (GC, Agilent 6890N) [44]. At the feed side, nitrogen was introduced under 0.4 MPa and 298 K. Helium was used as the sweeping gas with a flow rate of ca. 10 mL/min.

Scanning electron microscopy (SEM) for membranes and particles was performed using a FEI Nanosem 430 field emission scanning electron microscopy (FE-SEM) at an acceleration voltage of 10~20 kV (Hillsboro, OR, USA). X-ray diffraction (XRD) data were obtained on a Rigaku D-max2500v/pc X-ray diffractometer using Cu Kα radiation in the range of 2θ = 5~50° at a scanning rate of 4°/min (Tokyo, Japan). UV-vis spectra were recorded by using a Jasco V-550 UV-vis spectrophotometer equipped with an integrated sphere (Easton, MA, USA). The composition of Si, Al, P, and metal along the membrane thickness was measured using electron probe microanalysis (EPMA-1600, Shimadzu, Kyoto, Japan) operated at 15 kV with a secondary electron resolution of 6 nm.

## 5. Conclusions

We have reported a novel strategy for manipulating continuous and well-defined AFI membranes on macroporous α-Al_2_O_3_ substrates in in situ crystallization. Based on our own results and literature data, it revealed that the use of a low molecular weight (LMW) chitosan layer as the structure-directing matrix resulted in the formation of single-layer AFI membranes, while double-layer AFI membranes were formed when a medium molecular weight (MMW) chitosan layer was employed, due to their difference in swelling ability and erodible rate in the acid precursor solution. The AFI membrane was composed of hexagonal prism-shaped crystals when aluminum isopropoxide (AIP) was used as the Al source, whereas spherical agglomerates of small crystals would be the membrane components if AIP is replaced by pseudo-boehmite. The coverage, denseness, and orientation of all of the AFI membranes could be further optimized by adjusting the water content in the precursor solution by fixing both the substrate setting (facing downwards) and the ratio of SiO_2_/Al_2_O_3_ (0.15). Besides, the addition of Si or metallic elements into the AFI framework would reduce the aspect ratio of hexagonal prism-shaped AFI crystals, usually leading to the formation of thinner *c*-oriented AFI membranes.

Control experiments confirmed that the chitosan layer should be indispensable for the continuity and orientation of AFI membranes on porous α-Al_2_O_3_ substrates in the in situ crystallization. The single-layer AFI membrane could be induced by using a thin layer of LMW chitosan due to its smaller swelling area and faster dissolution rate, whereas the double-layer AFI membrane would be fabricated under the structure-directing function of a MMW chitosan layer with a larger swelling area and a lower dissolution rate. The established methodology in this study has provided the platform to fabricate continuous and well-defined AFI membranes with single and double-layers of hexagonal prisms or crystal agglomerates, which will have application potential in the separation of gas/liquid mixtures, membrane catalysis, and microelectronic devices.

## Supplementary Materials

The supporting Information (Figures S1~S7) are available online.
