# Supplementary material for: Efficient Manipulation of Continuous AFI-Type Aluminophosphate Membranes with Distinctive Microstructures on Macroporous α-Al2O3 Substrates
Source: Molecules. 2018 May 9;23(5):1127. doi: 10.3390/molecules23051127 (PMC6099800; doi:10.3390/molecules23051127)
Supplement: Supplementary file 1 [file molecules-23-01127-s001.pdf]

Supporting Information

Efficient Manipulation of Continuous AFI-type Aluminophosphate Membranes with Distinctive Microstructures on Macroporous α-Al_2O_3 Substrates

Luwei Geng, Hongfeng Dong*, Xiufeng Liu, Baoquan Zhang

School of Chemical Engineering and Technology, Tianjin University, Tianjin 300350, China

*Corresponding author, hongfeng_007@126.com

Postal address: Room 244, Building 52, Bei-Yang-Yuan Campus of Tianjin University,

Haihe Education Park, Jinnan District, Tianjin 300350, China

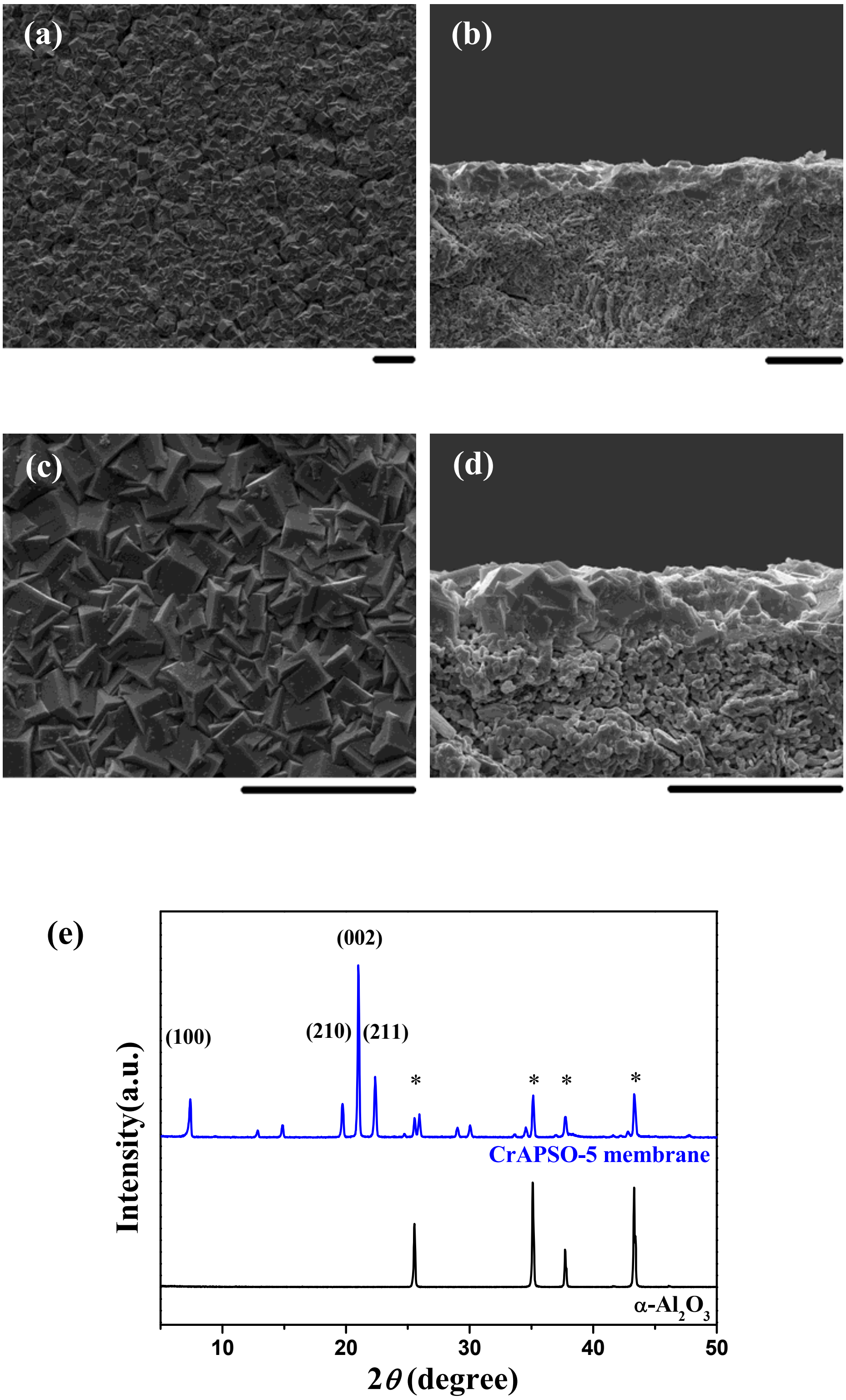

Figure S1. (a, c) Top-view and (b, d) cross-sectional-view SEM images together with (e) the corresponding XRD pattern of the CrAPSO-5 membrane induced by a layer of LMW chitosan using aluminum isopropoxide (AIP) as the Al source. The asterisks mark the peaks originating from the α-Al2O3 substrate. Scale bars correspond to 20 μm in SEM images.

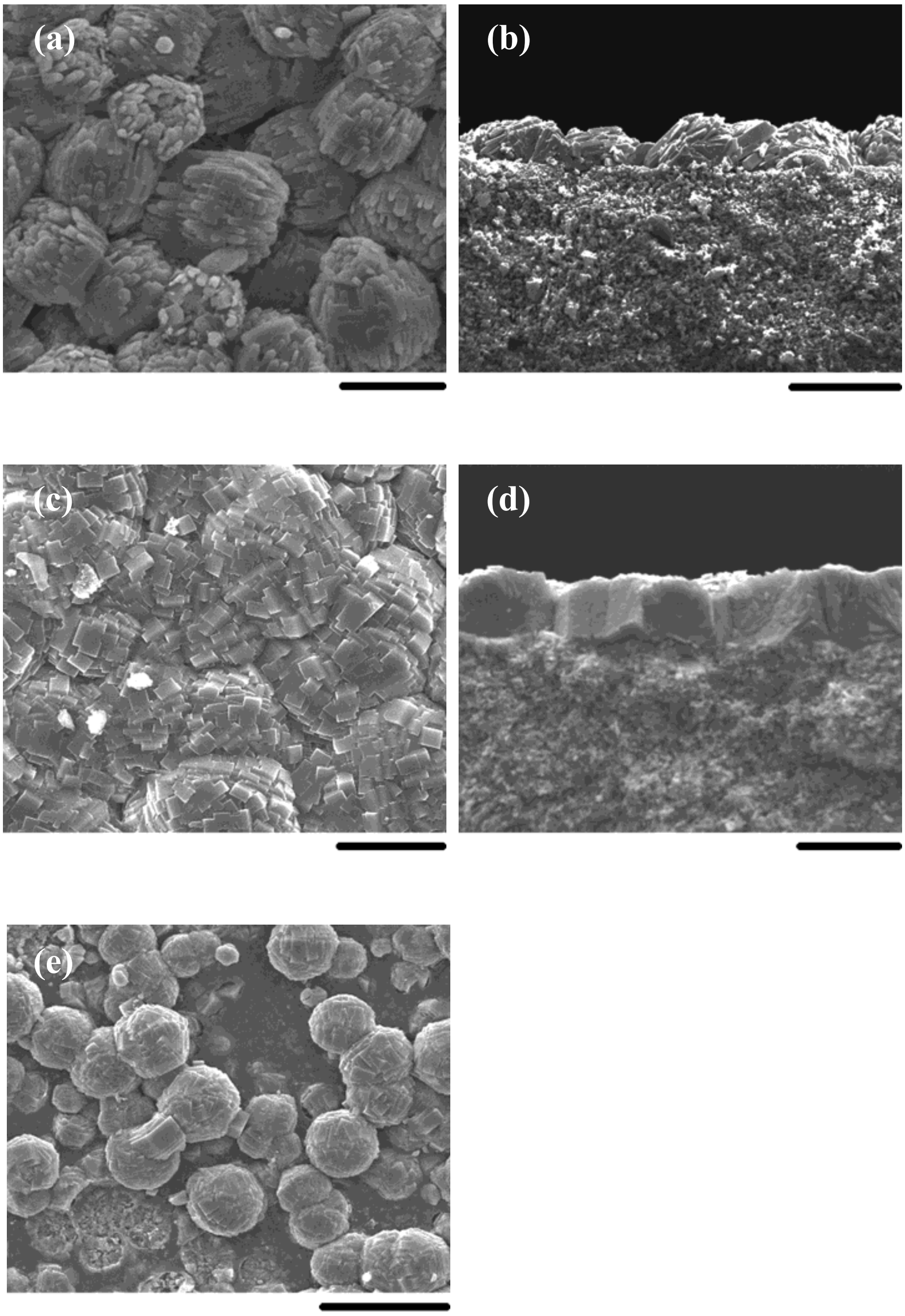

Figure S2. SEM images of SAPO-5 membranes induced by a layer of LMW chitosan at pH = 4.5 (a, b), 6.0 (c, d), and 7.0 (e), using pseudo-boehmite as the Al source. Scale bars correspond to 20 μm.

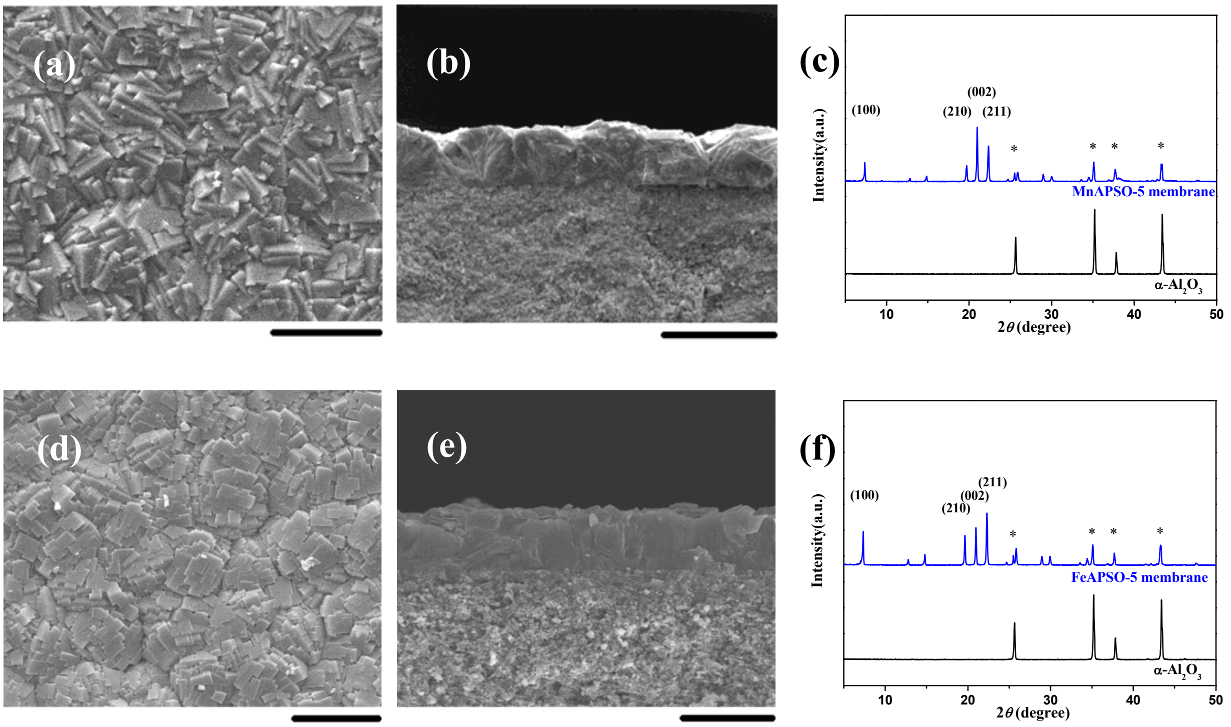

Figure S3. Top-view and cross-sectional SEM images together with the corresponding XRD pattern of (a, b, c) MnAPSO-5 and (d, e f) FeAPSO-5 membranes induced by a layer of LMW chitosan using pseudo-boehmite as the Al source. Scale bars correspond to 20 μm in SEM images.

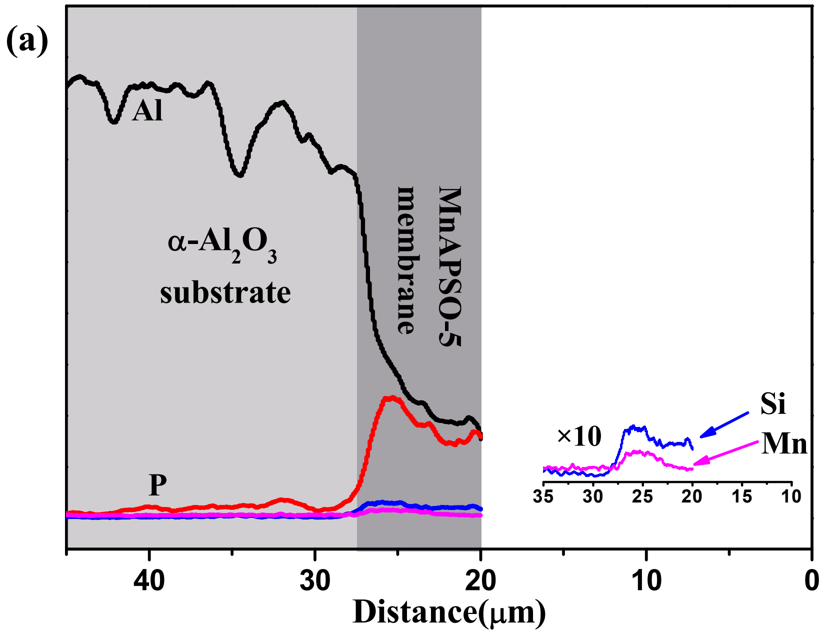

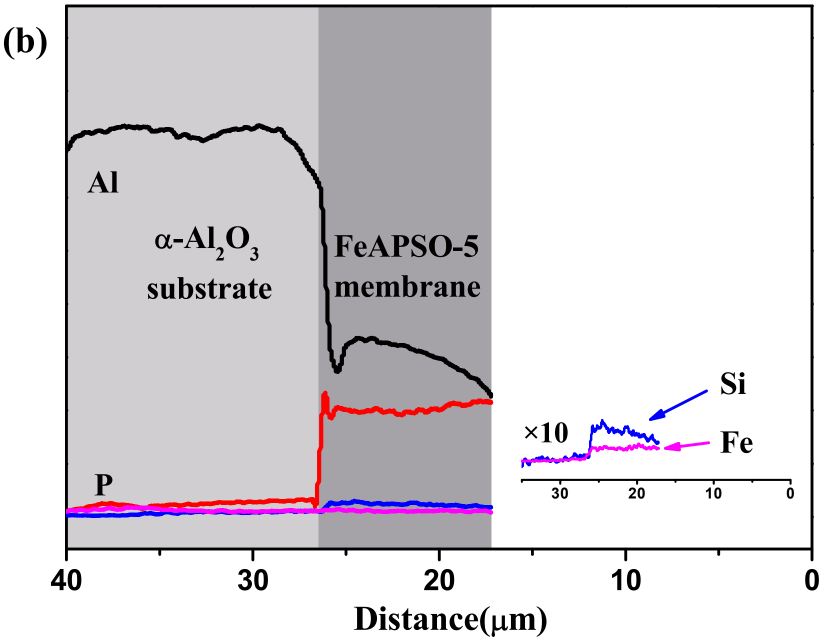

Figure S4. EPMA measurements of (a) MnAPSO-5 and (b) FeAPSO-5 membranes induced by a layer of LMW chitosan using pseudo-boehmite as the Al source.

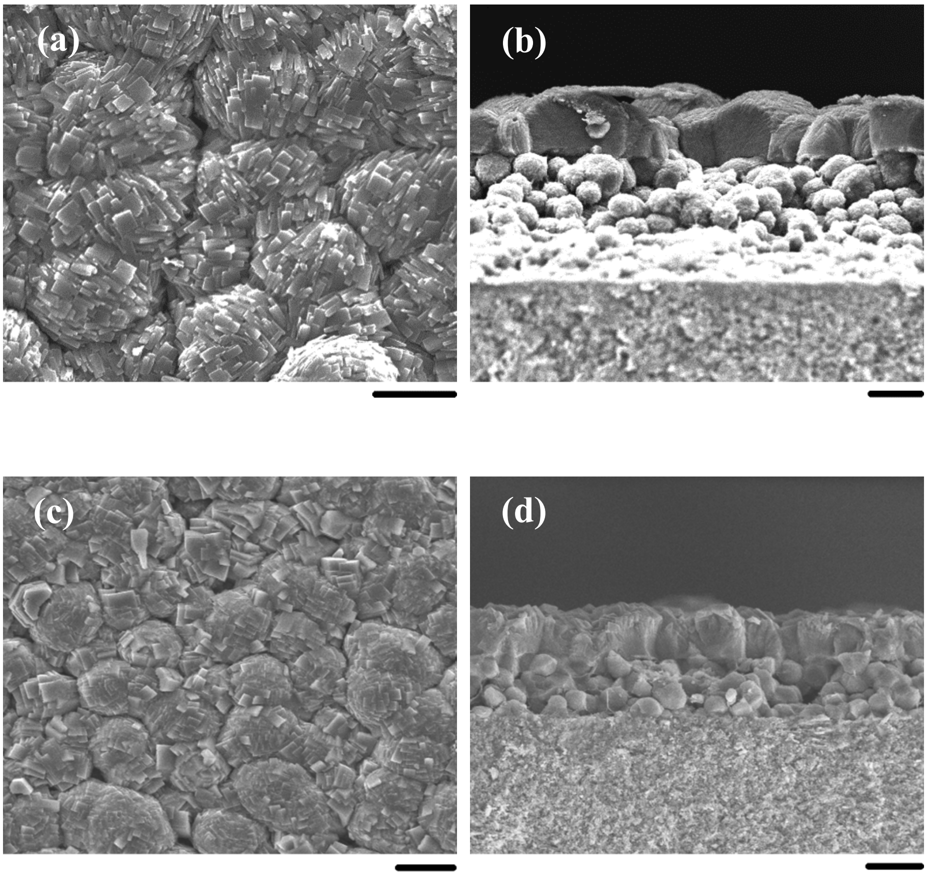

Figure S5. Top-view and cross-sectional-view SEM images of (a, b) MnAPSO-5 and (c, d) CoAPSO-5 membranes induced by a layer of MMW chitosan using pseudo-boehmite as the Al source. Scale bars correspond to 50 μm.

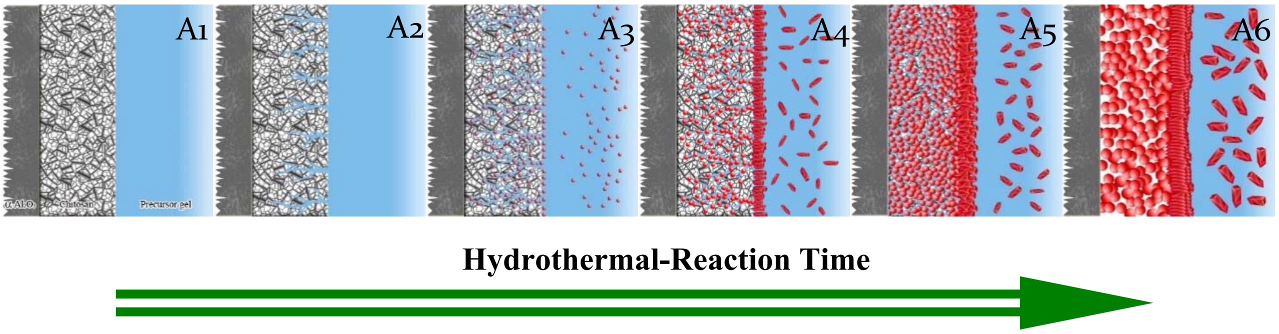

Figure S6. Proposed scheme for the chronological evolution of preferentially *c*-oriented AFI membranes on α-Al2O3 supported layers of MMW chitosan (A1-A6).

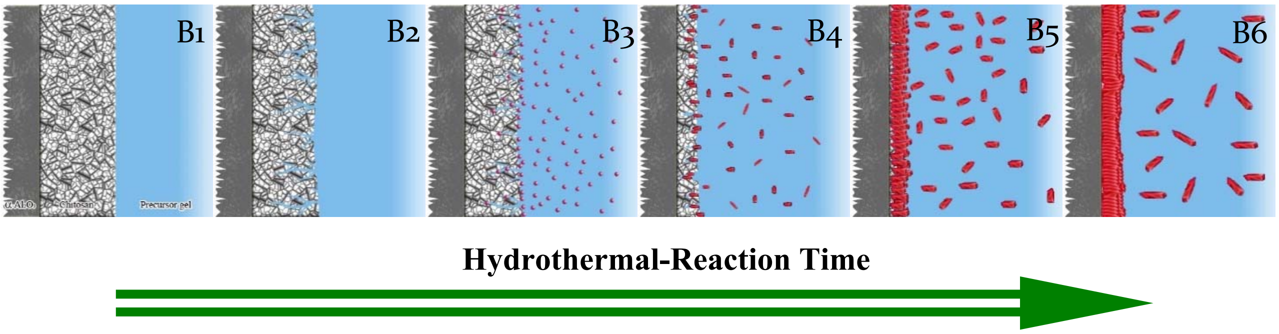

Figure S7. Proposed scheme for the chronological evolution of preferentially *c*-oriented AFI membranes on α-Al2O3 supported layers of LMW chitosan (B1-B6).
